# Clinical and genetic characteristics of maturity‐onset diabetes of the young type 13: A systematic review of the literature

**DOI:** 10.1111/1753-0407.13520

**Published:** 2023-12-14

**Authors:** Yaning Chen, Xiaodong Hu, Mingwei Zhao

**Affiliations:** ^1^ Department of Endocrinology The Sixth Medical Center of Chinese PLA General Hospital Beijing China

**Keywords:** gene mutation, *KCNJ11*, Kir6.2, MODY13

## Abstract

**Objective:**

Maturity‐onset diabetes of the young type 13 (MODY13), a rare type of monogenic diabetes, is often misdiagnosed as type 1 or type 2 diabetes. To improve early diagnosis and precise treatment, we performed a systematic review and analysis of the literature about MODY13.

**Methods:**

PubMed, Cochrane, Embase, China National Knowledge Infrastructure (CNKI), Chinese BioMedical (CBM) Literature Database, and Wanfang Database were searched using the following search terms: “MODY13,” “*KCNJ11* maturity‐onset diabetes of the young,” “*KCNJ11*‐MODY,” “maturity‐onset diabetes of the young type 13,” and “neonatal diabetes mellitus *KCNJ11*.” The demography, clinical characteristics, and gene mutations of patients were expressed with descriptive statistical methods.

**Results:**

A total of 33 reports were included in this study, including 75 patients and 28 types of mutations. Thirty‐six patients were male. The mean onset age was 25.20 ± 15.26 years. The averages of recorded body mass index, glycated hemoglobin (HbA1c), and fasting C‐peptide were 23.45 ± 4.56kg/m^2^, 10.07 ± 1.96%, and 0.31 ± 0.23nmol/L, respectively. Most of the mutation sites were located in the cytosolic region of N‐ and C‐terminal domains of Kir6.2. Seven patients were reported to have diabetic chronic complications.

**Conclusion:**

MODY13 was diagnosed later than other types of MODY and was associated with low fasting C‐peptide. Mutation sites of MODY13 were mostly concentrated in N‐ and C‐terminal intracellular domains. The majority of *KCNJ11* gene mutations causing MODY 13 were from G to A. The incidence rates of chronic complications were lower than type 1 and type 2 diabetes.

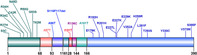

## Graphical abstract

Highlights

- This is the first article to summarily describe clinical and genetic characteristics of maturity‐onset diabetes of the young type 13 (MODY13).
- MODY13 was diagnosed later than other types of MODY and was associated with low fasting C‐peptide.
- Mutation sites of MODY13 were mostly concentrated in N‐ and C‐terminal intracellular domains.
- The incidence rates of chronic complications were lower than type 1 and type 2 diabetes.

## 1 INTRODUCTION

Maturity‐onset diabetes of the young (MODY) is caused by mutations in genes involved in the development and function of β‐cells.^1^ It is the most common type of monogenic diabetes in clinical practice, accounting for 1%–3% of all diabetes.^2^ Due to similar and overlapped clinical features, MODY is frequently misdiagnosed as type 1 diabetes mellitus (T1DM) or as type 2 diabetes mellitus (T2DM).So the prevalence of MODY may be underestimated. Many literatures mentioned that 14 types of genes had been confirmed to be associated with MODY, including *HNF4α, GCK, HNF1α, PDX1, HNF1β, NEUROD‐1, KLF11, CEL, PAX4, INS, BLK, ABCC8, KCNJ11*, and *APPL1*，named as MODY 1–14.^3^ Experts now prefer to simply using the gene names for clarity, such as *KCNJ11* diabetes (or *KCNJ11*‐MODY, but in this paper we still use MODY13).With the development of clinical molecular diagnostic technologies, the typing of MODY is changing. Broome et al^2^ summarized that mutations in more than 15 genes were associated with MODY. They suggested that experts should carefully review the variants in *BLK*, *KLF11*, *NEUROD1*, *PAX4*, and *PDX1*reported by genetic testing companies before these mutations were verified to be pathogenic and result in monogenic diabetes. Own^4^ reported 16 genes associated with MODY, including *BLK, PAX4*, and *KLF11*, but their genetic evidence was not well documented. Laver et al^5^ also demonstrated by variant— and gene‐level genetic evidence that *BLK*, *KLF11*, or *PAX4* was not a cause of MODY, and they proposed that these three genes should be removed from MODY diagnostic genetic testing.

MODY13, first put forward in 2012, was caused by mutations in potassium channel rectifying subfamily J (*KCNJ11*). Mutations in *KCNJ11* can also cause neonatal diabetes mellitus (NDM), which occurs mainly in the first 6 months of age and rarely between 6 months and 1 year of age.^6^ The prevalence of MODY13 in MODY population is <1%.^7^ To date, almost all studies on MODY13 are case reports, and clinical and genetic characteristics of MODY13 have not been studied. In order to better understand MODY13, we systematically reviewed and analyzed the literature of reported MODY13 in both English and Chinese.

## 2 METHODS

The protocol of this systematic review has been pre‐registered with International Prospective Register of Systematic Reviews (PROSPERO) (No. CRD42022382068) (https://www.crd.york.ac.uk/prospero/display_record.php?ID=CRD42022382068). We conducted this systemic review in accordance with the Preferred Reporting Items for Systematic Reviews and Meta‐Analyses (PRISMA) reporting guidelines.

### 2.1 Data sources and searches

PubMed, Cochrane, Embase, China National Knowledge Infrastructure (CNKI), Chinese BioMedical (CBM) Literature Database, and Wanfang Database were searched for studies reporting the cases of MODY13 from the date of inception to 19 December 2022. The search terms included “MODY13,” “*KCNJ11* maturity‐onset diabetes of the young,” “*KCNJ11*‐MODY,” “maturity‐onset diabetes of the young type 13,” and “neonatal diabetes mellitus *KCNJ11*.” The search strategy was shown in supplementary material Table S1. Although the conception of MODY13 was proposed in 2012, some patients reported before that could be diagnosed with MODY13 based on clinical features and genetic mutations according to diagnostic criteria. All titles and abstracts searched were imported to EndNote software, version 20.

### 2.2 Study selection

The typical clinical criteria for the diagnosis of MODY included (a) early‐onset of diabetes at <25 years of age; (b) diabetes in at least two or ideally three family members; (c) not requiring insulin at least 5 years after diagnosis; and (d) absence of obesity or diabetic ketoacidosis.^8^ But using these criteria, only 48% of MODY cases were identified, so they are not sufficiently sensitive to be used alone in clinical practice.^9^

All the enrolled studies met all the following criteria: (a) the diagnosis of MODY13 was confirmed by genetic test and the mutated loci were described; (b) the language of the literature was English or Chinese; and (c) some clinical characteristics of patients were included.

Studies were excluded if they met any of the following criteria: (a) studies on animal or in vitro experiments; (b) double—and triple‐heterozygosity; and (c) patients with only the mutated loci supplied.

Eligible articles identified after the title and abstract review were read in full text, and the reference lists were searched for additional literatures. This step was carried out by two independent reviewers (Y.N.C. and M.W.Z.), and any disagreements were resolved by a third reviewer (X.D.H.).

### 2.3 Data collection process

Y.N.C. independently extracted all the data. X.D.H. and M.W.Z. also performed the data extraction independently. Any disagreements were resolved by coming to a consensus.

Demographic, clinical, genetic, and treatment data were extracted using standardized tables. The diagnostic criteria for diabetes follow the 1999 World Health Organization Expert Committee Report on Diabetes. MODY13 was diagnosed by clinical characteristics, family history, and genetic mutation. Body mass index (BMI) was categorized as follows: underweight (<18.5 kg/m^2^), normal weight (18.5–24 kg/m^2^), overweight (24–28 kg/m^2^), and obese (≥28 kg/m^2^). Detailed clinical data and genetic mutations are summarized in Supplementary material, Table S2.

### 2.4 Statistics

There was obvious heterogeneity of the included reports, and we cannot perform a meta‐analysis. Therefore, we mainly conducted a narrative synthesis. Descriptive data were shown as observed counts and percentages and continuous data as means ± SDs. Student *t* test was used to identify difference among two independent groups. A two‐sided *p* value <.05 was considered statistically significant. Statistical analyses were performed using IBM SPSS Statistics version 25.

## 3 RESULTS

**FIGURE 1:**
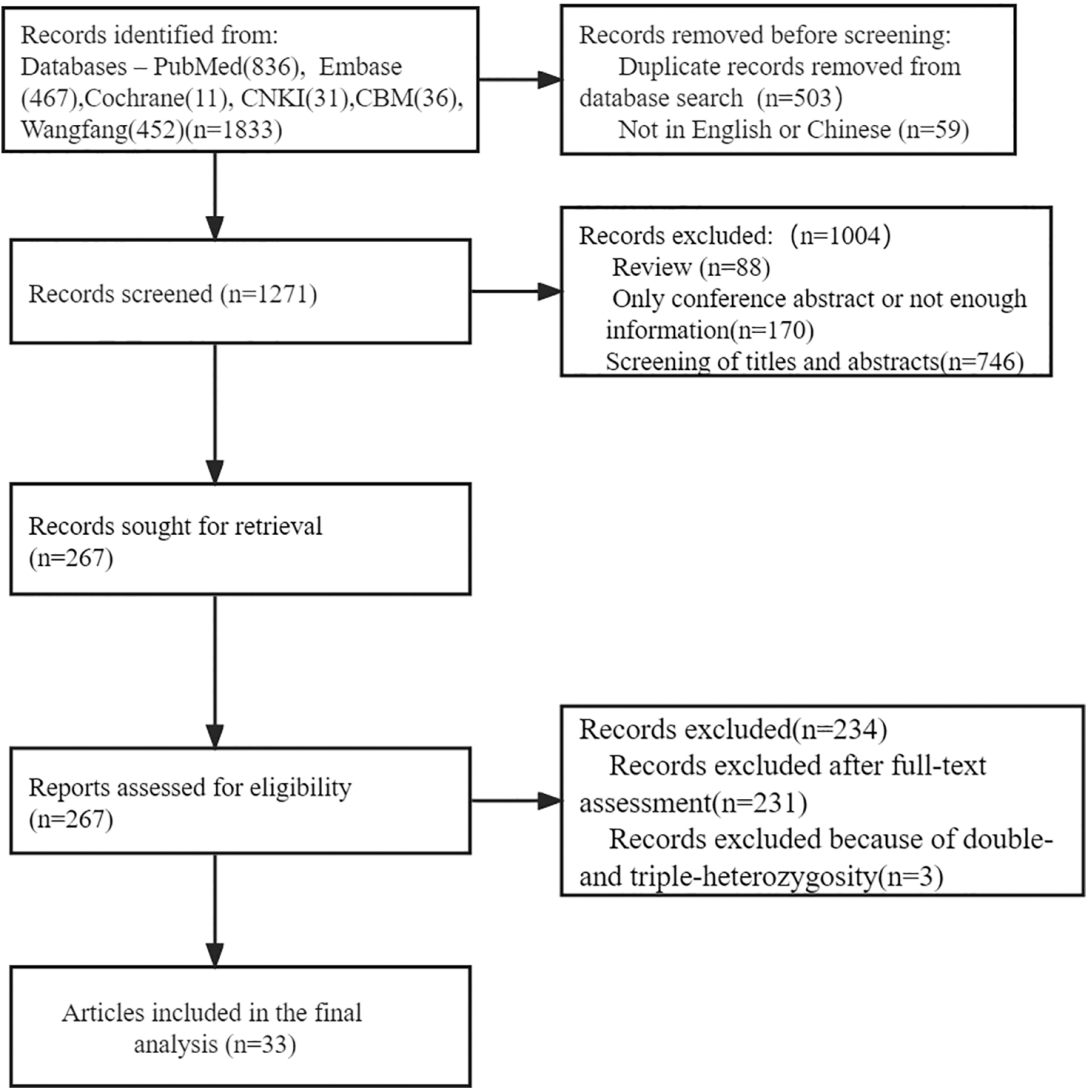
Flow chart for searching literatures.

A total of 1833 articles were searched on PubMed, Cochrane, Embase, CNKI, CBM Literature Database, and Wanfang Database. Before the process of screening, 562 articles were excluded because of duplication and other languages. After the process of screening and assessing, 1238 articles were excluded. Thirty‐three articles were included after being assessed for eligibility. The detailed procedure is shown in Figure 1.

### 3.1 General data

A total of 33 publications included 75 eligible patients of 44 families from 14 countries on four continents. Among 44 families, 13 were from China, six from Italy, four from the United Kingdom, four from Turkey, and 10 respectively from Japan, Israel, Spain, Cyprus, Iran, France, India, Brazil, Australia, and Portugal. Among 75 patients, 39 were from Asia (39/75, 52%), 37 from Europe (27/75, 36%), and two respectively from Oceania and South America. In seven cases or families where they came from was not mentioned.

### 3.2 Clinical features

Clinical data of the patients were shown in Table 1. Among 75 patients, the gender of two patients was unavailable. Thirty‐six patients were male (36/73, 49.3%) and 37 patients were female (37/73, 50.7%). The age at onset was recorded in 70 patients, with a mean age of 25.20 ± 15.26 years. Thirty‐nine patients (39/70, 55.71%) were diagnosed with diabetes before the age of 25 years. Nine patients (9/70, 12.86%) developed diabetes before the age of 10 years. Four patients (4/75, 5.33%) were mentioned to have experienced diabetic ketoacidosis at the time of diagnosis. The duration of diabetes was recorded in 46 patients with an average of 16.03 ± 15.07 years. Eighteen patients had diabetes for <10 years, 13 patients (13/46, 28.26%) between 10 and 20 years, and 15 patients (15/46, 32.61%)>20 years. The history of diabetes of two patients was 55 years. BMI was recorded in 35 patients with an average value of 23.45 ± 4.56 kg/m^2^. Seven patients were underweight (<18.5 kg/m^2^), and 15 patients had normal weight (18.5–24 kg/m^2^), including a patient without the value of BMI but described as “normal weight.” Eight patients were overweight (24–28 kg/m^2^), including one patient who was mentioned as overweight but no specific values were available. Seven patients were obese (≥28 kg/m^2^).

**TABLE 1:** Clinical data of all patients with MODY 13.

	All recorded patients		Chinese patients		Non‐Chinese patients	
	*n*	Mean ± SD	*n*	Mean ± SD	*n*	Mean ± SD
Number of participants (M/F)	75 (36/37)		26 (14/12)		42 (18/22, 2 unknown)	
Age at onset (years)	70	25.20 ± 15.26	26	28.92 ± 13.75	38	22.25 ± 16.04
<25	39	14.01 ± 5.54	9	13.43 ± 4.50	26	13.79 ± 6.11
≥25	31	38.90 ± 11.73	17	37.12 ± 8.89	12	40.58 ± 15.65
Duration of diabetes (years)	46	16.03 ± 15.07	25	8.79 ± 8.69	20	23.98 ± 16.79
<10	18	2.84 ± 3.11	14	2.41 ± 2.96	4	4.38 ± 3.59
≥10 and <20	13	13.69 ± 2.78	7	12.86 ± 2.85	6	14.67 ± 2.58
≥20	15	33.87 ± 11.93	4	24 ± 3.56	10	37.40 ± 12.58
BMI (kg/m^2^)	35	23.45 ± 4.56	20	23.80 ± 4.30	15	22.98 ± 5.00
<18.5	7	17.68 ± 0.82				
≥18.5 and <24	15	21.71 ± 1.10				
≥24 and <28	8	25.67 ± 0.89				
≥28	7	30.49 ± 2.09				
FCP (nmol/L)	24	0.31 ± 0.23	17	0.30 ± 0.21	7	0.35 ± 0.27
HbA1c before adjustment (%)	18	10.07 ± 1.96	10	10.26 ± 1.72	8	9.85 ± 2.29
HbA1c after adjustment (%)	31	6.44 ± 0.89	12	6.48 ± 0.97	19	6.43 ± 0.87
Diabetic ketoacidosis at onset	4					
Islet‐associated antibodies	19	Negative				

Abbreviations: BMI, body mass index; FCP, fasting C‐peptide; HbA1c, glycated hemoglobin.

Fasting C‐peptide (FCP) was recorded in 24 patients with a mean of 0.31 ± 0.23 nmol/L, including one too low not to be measured and recorded as 0 in our study. Glycated hemoglobin (HbA1c) before adjusting the treatment was recorded in 18 patients with an average of 10.07 ± 1.96%, excluding cases without adjustment. HbA1c after transforming therapy was recorded in a total of 31 patients, including cases without adjustment, with a mean value of 6.44 ± 0.89%, and the HbA1c levels of 24 patients were not >7%. Islet‐associated antibodies were recorded as negative in 19 patients.

Among 758 patients, 26 patients (14 male/12 female) were from China, and 42 cases (18 male/ 22 female，gender unavailable in two cases) were from other countries (see Table 1). The mean age at onset was 28.92 ± 13.75 years in 26 Chinese and 22.25 ± 16.04 years in 38 cases from other countries. 34.62% of Chinese cases (9/26) and 68.4% of cases (26/38) from other countries were diagnosed with diabetes before the age of 25. Fifteen cases from other countries had a mean BMI of 22.98 ± 5.00 kg/m^2^ and the average BMI of 20 Chinese was 23.80 ± 4.30 kg/m^2^. There was no statistical difference between the two groups. The mean duration of diabetes was 8.79 ± 8.69 years in 25 Chinese and 23.98 ± 16.79 years in 20 patients from other countries, which were statistically significant. The level of FCP was 0.30 ± 0.21 nmol/L in 17 Chinese and 0.35 ± 0.27 nmol/L in seven patients from other countries.

### 3.3 Gene mutations

**FIGURE 2:**
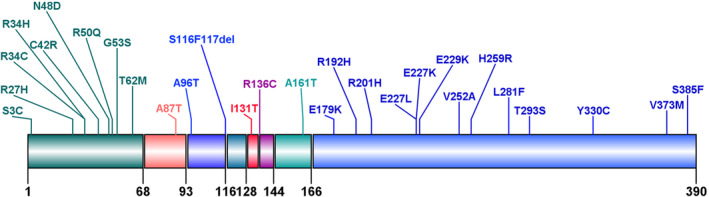
Reported mutation sites in Kir6.2 causing MODY13. MODY13, maturity‐onset diabetes of the young type 13.

The mutation profile of the patients was shown in Figure 2. Twenty‐eight types of mutations were identified, including substitutions (27/28, 96.43%) and small deletions (1/28, 3.57%). In terms of families, the most frequent mutation was E227K appearing in seven families (7/44, 15.91%), followed by E229K in five families (5/44, 11.36%) and A161T in three families (3/44, 6.82%). The same mutation caused NDM in 19 families. In terms of the number of patients diagnosed, 19 patients (17/75, 22.67%) had the E227K mutation, seven patients (7/75, 9.33%) had the E229K mutation, and five patients (5/75, 6.67%) had the C42R mutation. Nine types of mutations (9/28, 32.14%) were located in the cytosolic region of N‐terminal domains. Thirteen types of mutations (13/28, 46.43%) lay in the cytosolic region of C‐terminal domains. The other six mutation types were in transmembrane domain 1, extracellular (amino acid 94–116) domain, H5 domain, pore‐forming domain, extracellular (amino acid 136–144) domain, and transmembrane domain 2, respectively. Mutation from guanine (G) to adenine (A) in DNA coding region appeared in 40 patients (40/62, 64.5%).

The similarities between two amino acids were quantified based on their polarity, molecular volume, and chemical composition.^10^ The values for properties in amino acid difference formula of 10 mutations were <50 and the values of 17 mutations were >50. FCP was 0.24 ± 0.23 nmol/L in nine patients the values for properties of whose mutations were < 50. FCP was 0.37 ± 0.23 nmol/L in 14 patients the values for properties of whose mutations were > 50. There was no statistical difference between the two groups (*p* > .05).

### 3.4 Treatment and chronic complications

The therapeutic regimens were documented in 59 patients. After adjusting for hypoglycemic drugs, 11 patients were treated with insulin alone or in combination with other hypoglycemic drugs. And 48 patients controlled blood sugar by sulfonylureas or other oral hypoglycemic or only diet. Twenty‐one patients successfully switched from insulin to sulfonylureas and they had good glycemic control. One patient changed sulfonylurea to insulin and controlled blood glucose well. The HbA1c levels of 10 patients who switched from insulin to sulfonylurea were recorded and decreased from 10.32 ± 2.00% before adjustment to 6.44 ± 0.77%.

In all the reports, seven patients (7/75, 9.33%) were recorded with chronic complications of diabetes. Four patients (4/75, 5.33%) had diabetic retinopathy. Three patients (3/75, 4.00%) were diagnosed with diabetic peripheral neuropathy by nerve conduction velocity. Diabetic kidney diseases happened to two patients (2/75, 2.67%).

## 4 DISCUSSION

Our study systematically reviewed the clinical features and genetic mutations of 75 patients diagnosed with MODY13. We summarized that patients with MODY13 had the following characteristics: (a) similar prevalence between males and females; (b) the onset age of nearly half of the patients >25 years old; (c) nonobese diabetes; (d) low FCP and different amino acid mutations did not affect the concentration of FCP; (e) mutation sites mostly concentrated in intracellular N‐ and C‐terminal domains of Kir6.2 and the majority of gene mutations causing MODY13 were from G to A; and (f) lower incidence rates of chronic complications than type 1 and type 2 diabetes.

*KCNJ11* gene at 11p15.1 contains only one exon and encodes a 390‐amino‐acid protein, namely inwardly rectifying potassium channel (Kir)6.2, with a molecular weight of 43.5 kDa.^11^ Kir6.2 is a typical inwardly rectifying potassium channel consisting of two transmembrane domains linked by a pore circle, intracellular amino, and carboxyl termini. Four Kir6.2 subunits and four sulfonylurea receptor (SUR1) subunits encoded by ATP‐binding cassette transporter subfamily C member 8 (*ABCC8*) constitute the ATP‐sensitive potassium (K_ATP_) channel in β‐cells. Kir6.2 subunits form the central tetrameric pore of K_ATP_ channel and SUR1 subunits are the regulatory subunits.^12^ Kir6.2 possesses binding sites for ATP and phosphatidylinositol 4,5‐bisphosphate, which inhibit and activate the channel, respectively.^13^, ^14^ In pancreaticβ‐cells, increased glucose metabolism will increase the ratio of ATP/ADP. Binding of ATP to K_ATP_ channel results in its closure, which leads to the opening of voltage‐gated Ca^2+^ channels, Ca^2+^ influx and the release of insulin.^15^ When an activating mutation happened in *KCNJ11*, K_ATP_ channel cannot be normally closed under the stimulation of glucose and the cell membrane remained hyperpolarized. Extracellular Ca^2+^ cannot flow inward and insulin cannot be secreted normally by β cells, which causes diabetes.^16^ Activating *KCNJ11* mutations together with activating *ABCC8* mutations can account for approximately 40% of NDM cases. In contrast, individuals diagnosed with monogenic diabetes outside of infancy are generally classified as having MODY12 by *ABBC8* and MODY13 by *KCNJ11*.^17^

MODY13 is an autosomal dominant form of diabetes caused by mutations in the *KCNJ11* gene, and the pathological basis is the dysfunction of pancreatic beta cells and absolute deficiency of insulin secretion. Most MODY13 patients were misdiagnosed as T1D or T2D at the beginning of the disease. Therefore, it was important to identify the clinical features of patients with MODY13 for early differential diagnosis.

Most patients with MODY are diagnosed before the age of 25 years. However, our study showed that 55.71% of patients developed diabetes before the age of 25 years, and 44.29% were diagnosed after the age of 25 years. Thus, MODY13 exhibited a slightly later age of onset than other types of MODY. The patients with MODY13 had relatively low FCP and it tends to be diagnosed in nonobese patients. Compared to those with T1D, MODY patients rarely presented with severe diabetic ketoacidosis, weight loss, and positive islet autoantibodies.^18^ They differ from the majority of patients with T2D in their young age of onset, nonobesity, and absence of insulin resistance.^4^

We found that most of the mutant sites of MODY13 were located in intracellular amino and carboxyl termini, which is similar to the reported mutation sites of Kir6.2 and can cause transient and permanent neonatal diabetes.^19^ The ATP‐binding site on Kir6.2 is located at the intracellular interface between two Kir6.2 subunits, close to the large cytosolic loop of SUR1.^20^ Martin GM et al^21^ discovered by cryoelectron microscopy that the major part of the binding pocket was contributed by the C terminus of one subunit, including a helical segment consisted of residues Y330, F333, and G334 and a β‐sheet containing residues K185 and I182. In addition, the N‐terminal N48 and R50 of the adjacent subunit interacted with ATP. When ATP interacted with Kir6.2, the broader interaction between various N and C‐domains played an important role in closing the pore of K_ATP_ channel.^15^, ^22^ So mutations in intracellular amino and carboxyl domains affected the affinity of ATP to the K_ATP_ channel. Many literatures have also demonstrated by in vitro experiments how mutations in *KCNJ11* that resulted in MODY13 were the reduced sensitivity of K_ATP_ to ATP.^16^, ^23^, ^24^, ^25^, ^26^

Sulfonylureas bind to sulfonylurea receptors that are tightly linked to potassium channels and stimulate insulin secretion by the way of bypassing the metabolic steps or binding to and closing the channel directly.^20^ Heterozygous activating mutations in*KCNJ11*can cause NDM and sulfonylureas, which can convert subcutaneous injection to convenient oral hypoglycemic drugs and improve patients' compliance, were an effective choice for those patients. However, not all the patients with NDM caused by *KCNJ11* mutations responded well to sulfonylureas. It has been reported that the effect of sulfonylureas on NDM may be related to the mutation sites of *KCNJ11*. For example, patients carrying C166Y, I296L, L164P, or T293N mutations cannot be successfully converted from insulin to sulfonylureas.^27^ Many studies found that oral sulfonylureas can also provide good glycemic control in MODY13 caused by the *KCNJ11* mutation. The level of insulin became significantly higher after starting sulfonylureas than before.^28^, ^29^ Our study showed that 21 patients had good glucose control after switching from insulin to sulfonylureas. The patient with *KCNJ11* S116117del changed sulfonylureas to insulin and blood glucose was controlled better. In that literature, the authors found that the mutation made the channel compressed, preventing K^+^ from flowing freely.^16^

Chronic complications of diabetes increase the financial burden of patients and are an important cause of death and disability. The longer the duration of diabetes, the higher the risk of chronic complications. In our study, one patient with a 55‐year history was not reported to have chronic complications. Patients with MODY13 did not face an increased risk of microvascular complications of diabetes, similar to MODY2,^30^ but lower than MODY1 and MODY3, both of which are prone to diabetes‐related vascular complications.^31^ The incidence of microvascular complications was significantly lower in MODY13 than in T1DM and T2DM. For T2DM, the early‐onset group had a higher prevalence of diabetic retinopathy and a lower prevalence of diabetic peripheral neuropathy than the late‐onset group.^32^ In the future, we should pay more attention to screening for diabetic chronic complications in MODY13 to learn more about the incidence of chronic complications. The low incidence of microvascular complications in MODY13 may also be related to the small number of cases and incomplete documentation in the literature.

Our study has several limitations. First, the clinical characteristics of some patients were incomplete, which may have resulted in some bias in our study. Second, some clinical manifestations were difficult to analyze because of the low incidence of MODY13 and the small number of reported cases.

In conclusion, our study showed that MODY13 was diagnosed later than other types of MODY, and mutation sites were mostly concentrated in N‐ and C‐terminal intracellular domains. Sulfonylureas were an effective therapy for MODY13. According to clinical and genetic characteristics of MODY13, we can better distinguish MODY13 from T1D and T2D, which is helpful to guide the precise treatment and judge the prognosis of the disease.

## CONFLICT OF INTEREST STATEMENT

The authors declare that there is no conflict of interest.

## Supporting information


**Table S1.** Literature and strategy.

**Table S2.** Clinical characteristics and gene mutations of all patients with maturity‐onset diabetes of the young type 13 (MODY 13).

## Data Availability

The data generated during and/or analyzed during the current study are available from the corresponding authors on reasonable request.

## References

[1] Fajans SS , Bell GI . MODY: history, genetics, pathophysiology, and clinical decision making. Diabetes Care. 2011;34(8):1878‐1884. doi:10.2337/dc11-0035 21788644 PMC3142024

[2] Broome DT , Pantalone KM , Kashyap SR , Philipson LH . Approach to the patient with MODY‐monogenic diabetes. J Clin Endocrinol Metab. 2021;106(1):237‐250. doi:10.1210/clinem/dgaa710 33034350 PMC7765647

[3] Firdous P , Nissar K , Ali S , et al. Genetic testing of maturity‐onset diabetes of the young current status and future perspectives. Front Endocrinol (Lausanne). 2018;9:253. doi:10.3389/fendo.2018.00253 29867778 PMC5966560

[4] Owen KR . Monogenic diabetes in adults: what are the new developments? Curr Opin Genet Dev. 2018;50:103‐110. doi:10.1016/j.gde.2018.04.006 29734081

[5] Laver TW , Wakeling MN , Knox O , et al. Evaluation of evidence for pathogenicity demonstrates that BLK, KLF11 and PAX4 should not be included in diagnostic testing for MODY. Diabetes. 2022;71:1128‐1136. doi:10.2337/db21-0844 35108381 PMC9044126

[6] Beltrand J , Busiah K , Vaivre‐Douret L , et al. Neonatal Diabetes Mellitus. Front Pediatr. 2020;8:540718. doi:10.3389/fped.2020.540718 33102403 PMC7554616

[7] Urakami T . Maturity‐onset diabetes of the young(MODY): current perspectives on diagnosis and treatment. Diabetes, Metab Syndr Obes. 2019;12:1047‐1056. doi:10.2147/DMSO.S179793 31360071 PMC6625604

[8] Tattersall RB . Mild familial diabetes with dominant inheritance. Q J Med. 1974;43(170):339‐357. 4212169

[9] Shields BM , Hicks S , Shepherd MH , Colclough K , Hattersley AT , Ellard S . Maturity‐onset diabetes of the young (MODY): how many cases are we missing? Diabetologia. 2010;53(12):2504‐2508. doi:10.1007/s00125-010-1799-4 20499044

[10] Grantham R . Amino acid difference formula to help explain protein evolution. Science. 1974;185(4154):862‐864. doi:10.1126/science.185.4154.862 4843792

[11] Inagaki N , Gonoi T , Clement JP , et al. Reconstitution of IKATP: an inward rectifier subunit plus the sulfonylurea receptor. Science. 1995;270(5239):1166‐1170. doi:10.1126/science.270.5239.1166 7502040

[12] Babenko AP , Aguilar‐Bryan L , Bryan J . A view of SUR/KIR6.X, K_ATP_channels. Annu Rev Physiol. 1998;60:667‐687. doi:10.1146/annurev.physiol.60.1.667 9558481

[13] Cook DL , Hales CN . Intracellular ATP directly blocks K^+^ channels in pancreatic β – cells. Nature. 1984;311(5983):271‐273. doi:10.1038/311271a0 6090930

[14] Shyng SL , Nichols CG . Membrane phospholipid control of nucleotide sensitivity of K_ATP_ channels. Science. 1998;282(5391):1138‐1141. doi:10.1126/science.282.5391.1138 9804554

[15] Antcliff JF , Proks P , Sansom MSP , Ashcroft FM . Focus on Kir6.2: a key component of the ATP‐sensitive potassium channel. J Mol Cell Cardiol. 2005;38(6):927‐936. doi:10.1016/j.yjmcc.2005.01.007 15910877

[16] Liu LM , Nagashima K , Yasuda T , et al. Mutations in KCNJ11 are associated with the development of autosomal dominant, early‐onset type 2 diabetes. Diabetologia. 2013;56(12):2609‐2618. doi:10.1007/s00125-013-3031-9 24018988 PMC5333983

[17] Franco ED , Saint‐Martin C , Brusgaard K , et al. Update of variants identified in the pancreatic β‐cell KATP channel genes *KCNJ11* and *ABCC8* in individuals with congenital hyperinsulinism and diabetes. Hum Mutat. 2020;41(5):884‐905. doi:10.1002/humu.23995 32027066 PMC7187370

[18] Carlsson A , Shepherd M , Ellard S , et al. Absence of islet autoantibodies and modestly raised glucose values at diabetes diagnosis should lead to testing for MODY: lessons from a 5‐year pediatric Swedish National Cohort Study. Diabetes Care. 2020;43(1):82‐89. doi:10.2337/dc19-0747 31704690 PMC6925576

[19] Flanagan SE , Clauin S , Bellanné‐Chantelot C , et al. Update of mutations in the genes encoding the pancreatic beta‐cell K_ATP_ channel subunits Kir6.2(*KCNJ11*) and sulfonylurea receptor 1(ABCC8) in diabetes mellitus and hyperinsulinism. Hum Mutat. 2009;30(2):170‐180. doi:10.1002/humu.20838 18767144

[20] Pipatpolkai T , Usher S , Stansfeld PJ , Ashcroft FM . New insights into K_ATP_ channel gene mutations and neonatal diabetes mellitus. Nat Rev Endocrinol. 2020;16(7):378‐393. doi:10.1038/s41574-020-0351-y 32376986

[21] Martin GM , Kandasamy B , DiMaio F , Yoshioka C , Shyng SL . Anti‐diabetic drug binding site in a mammalian K_ATP_ channel revealed by Cryo‐EM. Elife. 2017;6:e31054. doi:10.7554/eLife.31054 29035201 PMC5655142

[22] Markworth E , Schwanstecher C . Schwanstecher M.ATP‐mediates closure of pancreatic beta‐cell ATP‐sensitive potassium channels by interaction with 1 of 4 identical sites. Diabetes. 2000;9(9):1413‐1418. doi:10.2337/diabetes.49.9.1413 10969823

[23] Li LT , Hou XG , Qin J , Liang K , Ren JM . A case report of maturity‐onset diabetes of the young 13. J Shandong Univ Health Sci. 2020;58(6):71‐75. doi:10.6040/j.issn.1671-7554.0.2019.1283

[24] Goonoo MS , Maclnerney RM . ‘Mody screening negative’: the importance of follow‐up. Diabet Med. 2019;36:81‐82. doi:10.1111/dme.13883

[25] Lo FS . Mutation screening of INS and KCNJ11 genes in Taiwanese children with type 1B diabetic onset before the age of 5 years. J Formos Med Assoc. 2018;117(8):734‐737. doi:10.1016/j.jfma.2018.01.002 29361385

[26] Ioannou YS , Ellard S , Hattersley A , Skordis N . KCNJ11 activating mutations cause both transient and permanent neonatal diabetes mellitus in Cypriot patients. Pediatr Diabetes. 2011;2(2):133‐137. doi:10.1111/j.1399-5448.2010.00743.x 21352428

[27] Babiker T , Vedovato N , Patel K , et al. Successful transfer to sulfonylureas in *KCNJ11* neonatal diabetes is determined by the mutation and duration of diabetes. Diabetologia. 2016;59(6):1162‐1166. doi:10.1007/s00125-016-3921-8 27033559 PMC4869695

[28] He BB , Li X , Zhou ZG . Continuous spectrum of glucose dysmetabolism due to the KCNJ11 gene mutation‐case reports and review of the literature. J Diabetes. 2021;13(1):19‐32. doi:10.1111/1753-0407.13114 32935446

[29] Qiu XP , Tu M , Chen Y , Qiu SL , Chen HJ , Tong Y . Maturity‐onset diabetes of the young type 13 in two families with KCNJ11 gene mutation: case report. Chin J Diabetes. 2021;29(6):410‐415. doi:10.3969/j.issn.1006-6187.2021.06.003

[30] Velho G , Blanché H , Vaxillaire M , et al. Identification of 14 new glucokinase mutations and description of the clinical profile of 42 MODY‐2 families. Diabetologia. 1997;40(2):217‐224. doi:10.1007/s001250050666 9049484

[31] Kant R , Davis A , Verma V . Maturity‐onset diabetes of the young: rapid evidence review. Am Fam Physician. 2022;105:162‐167. 35166506

[32] Huang JX , Liao YF , Li YM . Clinical features and microvascular complications risk factors of early‐onset type 2 diabetes mellitus. Curr Med Sci. 2019;39(5):754‐758. doi:10.1007/s11596-019-2102-7 31612393

