# Supplementary material for: Clinical and genetic characteristics of maturity‐onset diabetes of the young type 13: A systematic review of the literature
Source: J Diabetes. 2023 Dec 14;16(3):e13520. doi: 10.1111/1753-0407.13520 (PMC10925878; doi:10.1111/1753-0407.13520)
Supplement: Supplementary file 1 — Table S1. Literature and strategy. [file JDB-16-e13520-s002.docx]

| Database | Search Strategy |
| --- | --- |
| Pubmed | (‘MODY13’ OR ‘*KCNJ11* maturity-onset diabetes of the young’ OR ‘*KCNJ11*-MODY’ OR ‘maturity-onset diabetes of the young type 13’ OR （‘KCNJ11’ AND ‘MODY’） OR (‘neonatal diabetes mellitus’ AND ‘KCNJ11’)) |
| Cochrane library | ‘MODY13’ OR ‘*KCNJ11* maturity-onset diabetes of the young’ OR ‘maturity-onset diabetes of the young type 13’ OR （‘KCNJ11’ AND ‘MODY’） OR (‘neonatal diabetes mellitus’ AND ‘KCNJ11’ |
| Embase | (‘MODY13’ OR ‘*KCNJ11* maturity-onset diabetes of the young’ OR ‘*KCNJ11*-MODY’ OR ‘maturity-onset diabetes of the young type 13’ OR （‘KCNJ11’ AND ‘MODY’） OR (‘neonatal diabetes mellitus’ AND ‘KCNJ11’)):ti,ab,kw |
| China National Knowledge Infrastructure | ‘成人起病的青少年糖尿病13’ OR ‘KCNJ11-MODY’ OR ‘MODY13’ OR (‘新生儿糖尿病’ AND ‘KCNJ11’) OR ‘成人起病的青少年糖尿病 KCNJ11’ |
| Chinese BioMedical Literature Database | ‘成人起病的青少年糖尿病13’ OR ‘KCNJ11-MODY’ OR ‘MODY13’ OR (‘新生儿糖尿病’ AND ‘KCNJ11’) OR ‘成人起病的青少年糖尿病 KCNJ11’ |
| Wanfang Database | ‘成人起病的青少年糖尿病13’ OR ‘KCNJ11-MODY’ OR ‘MODY13’ OR (‘新生儿糖尿病’ AND ‘KCNJ11’) OR ‘成人起病的青少年糖尿病 KCNJ11’ |

**Table S1: Literature and strategy.**
