# Supplementary material for: Clinical and genetic characteristics of maturity‐onset diabetes of the young type 13: A systematic review of the literature
Source: J Diabetes. 2023 Dec 14;16(3):e13520. doi: 10.1111/1753-0407.13520 (PMC10925878; doi:10.1111/1753-0407.13520)
Supplement: Supplementary file 2 — Table S2. Clinical characteristics and gene mutations of all patients with maturity‐onset diabetes of the young type 13 (MODY 13). [file JDB-16-e13520-s001.docx]

| First author, year  Table S2: Clinical characteristics and gene mutations of all patients with MODY 13 | Family | Countr | Gender | Age at onset(y) | History of Diabetes(y) | BMI(kg/m^2^) | FCP  (nmol/L) | HbA1c(%) | treatment | Mutation (DNA) | Mutation  (Amino acid) | Adjusted treatment | HbA1c after adjustment |
| --- | --- | --- | --- | --- | --- | --- | --- | --- | --- | --- | --- | --- | --- |
| Liu YX, 2022 | 1 | China | F | 14 | 0.058 | 33.53 | 0.66 | 11.9 | Met+Ins | NM_000525.4:c.259G>A | p.A87T | Met |  |
| Monteior SS,2022 | 2 | Portugal |  | 29 |  |  | 0.73 |  |  | NM_000525:c.776A>G | p.H259R | NIHA | 8 |
| Song XJ,2022 | 3 | China | M | 19 | 0.01 | 21.88 | 0.216 | 12.9 | Ins | NM_000525:c.685G>A | p.E229K | GLIM |  |
|  |  | China | M | 45 | 0 | 26.78 | 0.63 | 9.4 |  | NM_000525:c.685G>A | p.E229K | GLIM |  |
| Maltoni G,2022 | 4 | Italy | M |  |  |  |  | 8.9 | Ins | c.685G>A | p.E229K | GLIM | 6.5 |
|  |  | Italy | M | 16 |  |  |  |  | Ins | c.685G>A | p.E229K | Ins |  |
| Yalcintepe S, 2021 | 5 | Turkey | F | 12 |  |  |  |  |  | NM_000525.3:c.481G>A | p.A161T |  |  |
|  | 6 | Turkey | M | 29 |  |  |  |  |  | NM_000525.3:c.1117G>A | p.V373M |  |  |
| He BB, 2021 | 7 | China  China  China | F | 13 | 3 |  | 0.107 | 10.1 | Ins | c.142A＞G  c.142A＞G  c.142A＞G | p.N48D | Gli | 7.2 |
|  |  |  | F | 16 | 2 |  | 0.35 | 8.6 | Ins |  | p.N48D | Gli | 6.2 |
|  |  |  | F | 36 |  |  |  |  | GLIM+Met |  | p.N48D | Gli+Met |  |
| Demirci DK,2021 | 8 | Turkey | M | 9.5 | 6.5 | 16.2 |  |  |  | c.1154C>T | p.S385F | SU | 6.2 |
| Chen YN,2021 | 9 | China | F | 28 | 11 | 24.68 | 0.5 | 10.3 | Met+Acar | NM_000525:c.406C>T | p.R136C | Gli+Met+Acar |  |
| Breidbart E, 2021 | 10 | Caucasian | F | 30 |  | OW |  |  |  | NM_000525:c.685G>A | p.E229K |  | 6.2 |
| Bonfanti R, 2021 | 11 | Italy | F | 7 |  |  |  |  |  |  | p.R50Q |  |  |
|  | 12 | Italy | M | 37 | 13 |  |  |  | SU |  | p.E179K | Diet |  |
|  | 13 | Italy | F | 14 |  |  |  |  | Ins |  | p.E227L | SU |  |
|  |  | Italy | F | 26 |  |  |  |  |  |  | p.E227L | SU |  |
|  | 14 | Italy | F |  |  |  |  |  |  |  | p.T293S | SU |  |
|  |  | Italy | M |  |  |  |  |  |  |  | p.T293S | Diet |  |
|  |  | Italy | M |  |  |  |  |  |  |  | p.T293S | Diet |  |
| Ates EA, 2021 | 15 | Turkey |  | 24 |  | 32 |  |  |  | NM_000525:c.841C>T | p.L281F |  |  |
| Qiu XP, 2021 | 16 | China | M | 12 | 2 | 25.8 | 0.65 |  | Ins | NM_000525.3:c.185C>T | p.T62M | Gli+Met |  |
|  |  | China | M | 36 | 10 |  |  |  |  | NM_000525.3:c.185C>T | p.T62M | Diet | 7 |
| Table S2. (Continued) |  | China | M | 59 | 10 |  |  |  |  | NM_000525.3:c.185C>T | p.T62M | Repaglinide | 6.5 |
|  | 17 | China | F | 26 | 14 | 21.23 | 0.43 |  |  | NM_000525.3:c.101G>A | p.R34H |  |  |
| Devaraka J, 2020 | 18 | UK | F | 14 | 26 |  | 0.43 | 15 | Ins |  | p.E227K | Gli | 6.2 |
| Li LT, 2020 | 19 | China | F | 15 | 0.058 | 18.37 | 0.23 | 9.6 | Ins | NM_000525.3:c.602 G>A | p.R201H | GLIM |  |
| Davis TME, 2019 | 20 | Australia | F | 14 |  | 28.7 | 0.66 | 6.8 | Met | 481G>A | p.A161T | Met+Ins |  |
| Goonoo MS, 2019 | 21 | UK | F | 14 | 16 | 21 | 0.289 |  | Ins | 481G>A | p.A161T | Gliclazide+Ins |  |
|  |  | UK | M | 18 |  |  |  |  |  | 481G>A | p.A161T |  |  |
| de Santana LS, 2019 | 22 | Brazil | F | 20 | 29 | N |  |  | Ins | NM_000525.3:c.286G>A | p.A96T | Ins+OHA | 6.4 |
| Ren L, 2019 | 23 | China | M | 13 | 0.08 | 17.4 | 0.23 | 9.3 |  | NM_000525:c.679 G>A | p.E227K | GLIM |  |
|  |  | China | M | 36 | 7 | 21.97 | 0.41 |  | Gliclazide+Met | NM_000525:c.679 G>A | p.E227K | Gliclazide+Met | 7.2 |
| Lo FS, 2018 | 24 | China | F | 2.83 | 8.5 |  | 0.16 | 12.7 | Ins | c.989A>G | p.Y330C | Gli | 7.8 |
| Day JO, 2017  Griscelli F, 2017  Ang SF, 2016  Liu LM, 2013 | 25 | UK  Unknown  China | F | 4 | 44 |  |  |  |  | NM_000525.3:c.392T>C | p.S3C |  |  |
|  | 26 |  | M | 13 | 38 |  |  |  |  |  | p.E227K |  |  |
|  | 27 |  | F | 16 | 27 | 23.1 |  |  |  |  | p.I131T | Ins+OHA | 8.1 |
|  | 28 | China  China | F  M | 40  29 | 16  2 | 24.5  32.4 | 0.13  0.1 |  | Ins  Ins | NM_000525.3:c.575G>A  NM_000525.3:c.575G>A | p.R192H  p.R192H | GLIM+Met  GLIM+Met | 5.6  5.5 |
|  |  | China  China | M  M | 32  30 | 2  7 | 28.4  25.2 |  |  | GLIM  GLIM | NM_000525.3:c.575G>A  NM_000525.3:c.575G>A | p.R192H  p.R192H | GLIM  GLIM |  |
|  | 29 | China | F | 33 | 17 | 26.2 |  | 7.8 | SU | NM_000525.3:c.348-353delCTTCTC | p.S116F117del | Ins | 5.2 |
|  |  | China | M | 46 | 27 | 23.6 | 0.13 |  | Ins | NM_000525.3:c.348-353delCTTCTC | p.S116F117del | Ins |  |
|  | 30 | China | M | 38 | 20 | 18.4 | 0.07 |  | Ins | NM_000525.3:c.80G>A | p.R27H | Gliclazide | 5.7 |
|  |  | China | M | 33 | 22 | 21 | 0.03 |  | Ins | NM_000525.3:c.80G>A | p.R27H | Gliclazide | 5.7 |
|  |  | China | F | 52 | 12 | 20.6 |  |  | Gliclazide | NM_000525.3:c.80G>A | p.R27H | Gliclazide |  |
|  |  | China | M | 32 | 0 | 21 |  |  | Gliclazide | NM_000525.3:c.80G>A | p.R27H | Gliclazide |  |
| Jahnavi S, 2013 | 31 | South India  Table S2. (Continued) | M | 52 |  |  |  |  |  | NM_000525.3:c.124T>C | p.C42R |  |  |
|  |  | South India | F | 50 |  |  |  |  |  | NM_000525.3:c.124T>C | p.C42R |  |  |
| Bonnefond A, 2012 | 32 | France | F | 16 | 3 | 18.4 |  |  | Ins | NM_000525.3:c.679G>A | p.E227K | Ins |  |
|  |  | France | M | 13 | 32 | 19.9 |  |  | OHA | NM_000525.3:c.679G>A | p.E227K | OHA | 7.1 |
|  |  | France | M | 17 | 15 | 21.7 |  |  | OHA/SU | NM_000525.3:c.679G>A | p.E227K | OHA/SU | 6.8 |
|  |  | France | M | 23 | 40 | 26.5 |  |  | OHA/SU | NM_000525.3:c.679G>A | p.E227K | OHA/SU | 8.6 |
|  |  | France | F | 59 | 0 | 23.7 |  |  | OHA/diet | NM_000525.3:c.679G>A | p.E227K | OHA/diet |  |
|  |  | France | M | 20 | 47 | 29 |  |  | OHA | NM_000525.3:c.679G>A | p.E227K | OHA | 6.2 |
|  |  | France | F | 47 | 26 | 21.5 |  |  | SU | NM_000525.3:c.679G>A | p.E227K | SU | 5.7 |
|  |  | France | F |  |  |  |  |  | Ins | NM_000525.3:c.679G>A | p.E227K | Ins |  |
| Abbasi F, 2012 | 33 | Iran | M | 2 | 12 |  | undetectable | 9.1 | Ins | NM_000525.3:c.679G>A | p.E227K | Gli | 6.6 |
|  |  | Iran | M | 15 | 20 |  |  | 9.2 | Ins | NM_000525.3:c.679G>A | p.E227K | Gli | 5.7 |
| Ioannou YS, 2011 | 34 | Cyprus | F | 74 |  |  |  |  |  | c.149 G>A | p.R50Q | SU |  |
| D’Amato E, 2008 | 35 | Italy | F | 26 | 13 |  |  |  | SU | NM_000525.2:c.679G>A,680A>T | p.E227L | SU |  |
|  |  | Italy | F | 14 | 55 | Not fat |  |  | SU | NM_000525.2:c.679G>A,680A>T | p.E227L | SU |  |
| Rica I, 2007 | 36 | Spanish | F | 13 |  |  |  |  |  |  | p.V252A |  |  |
| Landau Z, 2007 | 37 | Arab-Muslim | F | 9 | 8 | 21.7 | 0.1 | 9.9 | Ins |  | p.E227K | Gli | 6.1 |
|  |  | Arab-Muslim | M | 20 | 19 | 29.4 |  | 10.1 | Ins |  | p.E227K | Gli+Met | 7 |
| Flanagan SE, 2007 | 38 |  | F | 5 |  |  |  |  |  | NM_000525.3:c.157G>A | p.G53S |  |  |
|  | 39 |  | F | 48 |  |  |  |  |  | NM_000525.3:c.685G>A | p.E229K |  |  |
|  | 40 |  | M | 40 |  |  |  |  |  | NM_000525.3:c.100C>T | p.R34C |  |  |
|  | 41 |  | M | 23 |  |  |  |  |  | NM_000525.3:c.679G>A | p.E227K |  |  |
|  | 42 |  | M | 15 |  |  |  |  |  | NM_000525.3:c.685G>A | p.E229K | SU |  |
| Yorifuji T, 2005 | 43 | Japan | M | 22 |  | 17.8 | 0.23 |  | Ins | c.124T>C | p.C42R | Ins | 6.8 |
|  |  | Japan | F | 28 |  | 17.19 |  | 9.6 | Ins | c.124T>C | p.C42R | GLIM | 5.1 |
|  |  | Japan | M | 3 | 55 | Non-fat |  |  | Ins | c.124T>C | p.C42R | Chlorpropamide | 5 |
| Gloyn AL, 2005 | 44 | UK | F | 4.99 |  |  |  |  |  | c.157G>A | p.G53S | Ins | 6 |

**Abbreviations:** F, female; M, male; Ins, Insulin; OHA, oral hypoglycaemic agents, SU, sulfonylureas；GLIM, glimepiride；Gli, glibenclamide; NIHA, Non-Insulin Hypoglycemic Agents; met, metformin; acar, acarbose.

Table S2. (Continued)
